# Estimating the annual risk of infection with *Mycobacterium tuberculosis* among adolescents in Western Kenya in preparation for TB vaccine trials

**DOI:** 10.1186/s12879-019-4314-7

**Published:** 2019-08-02

**Authors:** Videlis Nduba, Anna H. van’t Hoog, Annefleur de Bruijn, Ellen M. H. Mitchell, Kayla Laserson, Martien Borgdorff

**Affiliations:** 1 Kenya Medical Research Institute, Centre for Respiratory Diseases Research (CRDR), PO Box 47855-00100, Nairobi, Kenya; 2 Academic Medical Center of the University of Amsterdam, Amsterdam, The Netherlands; 3 Amsterdam Institute for Global Health & Development, University of Amsterdam, Amsterdam, The Netherlands; 4 International Institute of Social Studies (ISS), Erasmus University Rotterdam, Rotterdam, The Netherlands; 5 Bill & Melinda Gates foundation, Seattle, USA

**Keywords:** ARTI, Tuberculosis, Adolescents, TST, Risk factors, TB vaccines

## Abstract

**Background:**

Adolescents are a prime target group for tuberculosis (TB) vaccine trials that include prevention of infection (POI). The BCG vaccine is given at birth and does not prevent TB infection. TB infection, a critical endpoint for POI vaccine trials would need to be documented to estimate sample sizes in target populations.

**Methods:**

Adolescents aged 12–18 years of age were enrolled in an area under continuous demographic surveillance. A tuberculin skin test (TST) survey was conducted as part of a study on TB prevalence and incidence. All adolescents got TSTs at enrolment and returned after 72 h for reading. A TST of ≥10 mm if HIV negative or ≥ 5 mm if HIV positive, was considered positive.

**Results:**

Of 4808 adolescents returning for TST readings (96% of those enrolled), mean age was 14.4 (SD 1.9), 4518(94%) were enrolled in school and 21(0.4%) gave a previous history of tuberculosis. Among adolescents with TST reactivity, the mean TST induration was 13.2 mm (SD 5.4). The overall prevalence of latent TB infection was 1544/4808 (32.1, 95% CI 29.2–35.1) with a corresponding annual risk of TB infection (ARTI) of 2.6% (95% CI 2.2–3.1). Risk factors for a positive TST included being male (OR 1.3, 95% CI 1.2,1.5), history of having a household TB contact (OR 1.5, 95% CI 1.2,1.8), having a BCG scar (OR 1.5,95% CI 1.2,1.8), living in a rural area (OR 1.4, 95% CI 1.1,1.9), and being out of school (OR 1.8, 95% CI 1.4,2.3).

**Conclusion:**

We conclude that the high TB transmission rates we found in this study, suggest that adolescents in this region may be an appropriate target group for TB vaccine trials including TB vaccine trials aiming to prevent infection.

**Electronic supplementary material:**

The online version of this article (10.1186/s12879-019-4314-7) contains supplementary material, which is available to authorized users.

## Background

Kenya ranks 10th out of 22 high TB burden countries globally [1]. The high prevalence of HIV in Kenya [1, 2] is a major contributing factor to TB incidence. Siaya County in Western Kenya has a high burden of tuberculosis and HIV with a TB case notification rate of 400/100,000 [3] and HIV prevalence of 15.1% [1].

Latent tuberculosis infection (LTBI) is the presence of *Mycobacterium tuberculosis* (MTB) in the body without signs and symptoms, or radiographic or bacteriologic evidence of tuberculosis (TB) disease [4]. One method to measure the trend of TB transmission is through repeated tuberculin surveys in order to estimate the trend of the prevalence of tuberculous infection and annual risk of tuberculous infection (ARTI). ARTI is used to measure the effectiveness of TB control programs. In addition, If TST conversion is a secondary endpoint for candidate vaccines preventing infection, ARTI estimates may also be used to inform the sample size of such trials.

Kenya has carried out several TST surveys to estimate the ARTI. These surveys were standardized by targeting primary school children aged 6–13 years and using the same TST technique [5–7]. The surveys were conducted in randomly selected districts and weighted for the underlying population distribution based on the most recent census. The ARTI estimates for Siaya district (where our present study was conducted) were 0.36, 1.10 and 1.45% for the surveys conducted in 1986–1990, 1994–1996 and 2004–2007 respectively with a mean age of 10 years in each sampled population indicating a rising ARTI over time [5–7].

Bacillus Calmette-Guerin (BCG), the TB vaccine given at birth, has not been shown to reliably prevent pulmonary tuberculosis in adolescents [8]. Adolescents may be prime candidates to receive new more effective TB vaccines because they are entering an age of steeply rising TB rates [9]. TB vaccine trials enrolling adolescents will need to measure efficacy using several endpoints, including TB disease incidence. Measuring the ARTI and the prevalence of infection in preparation for TB vaccine trials can give an indication of TB transmission in target communities. In this study, we aimed to estimate the annual risk of infection with MTB among 12–18 year olds. Some of the interim results of this study have previously been reported in an abstract at the Biennial infectious disease conference, Nairobi Hospital Convention Centre, Nairobi, Kenya, 2017.

## Methods

### Study design and sample size

The study area, Karemo Division with a population of 85,000 people, part of Siaya County is under a continuous health and demographic surveillance system (HDSS) [10]. The HDSS collects biannual data using household surveys. In brief, the study area was divided into 17 clusters of approximately equal population size. The clusters were randomly selected to give each adolescent an equal probability of participation. Eight out of 17 clusters were used for enrolment. In each cluster, HDSS data was used to identify households with adolescents; parents at these households were then approached to have their children participate in the study. The survey was conducted as part of a study on TB prevalence and incidence which used a prospective observational cohort design enrolling adolescents aged 12–18 years [11].

### Study procedures

#### Informed consent and clinical interviews

Parental consent was obtained at home by study staff prior to inviting adolescents to a mobile field site where minor assent was obtained prior to study participation. During enrolment, standard social demographic data were collected including age, sex, school enrolment status, parental social economic status, parental mortality, recent migration, and urban or rural residence. In addition, several clinical characteristics were collected including history of immunization with BCG, history of tuberculosis, BCG scar (we used the presence of a scar to stratify BCG status), weight and height. Study procedures have been described in more detail elsewhere [11]. The study received ethical approval of the Kenya Medical Research Institute and the US Centres for Disease Control and Prevention.

#### Tuberculin skin test and HIV testing

All enrolled adolescents were offered a tuberculin skin test (TST) for LTBI after they provided a clinical history and vital signs were recorded. The TST used 0.1 ml of tuberculin purified protein derivative (PPD) containing 5TU (tuberculin units) RT23 with Tween 80 (Statens Serum Institute, Copenhagen, Denmark) and was injected intradermally into the middle dorsal region of the right forearm using a disposable tuberculin syringe and G.26 needle. Adolescents were offered a TST after giving clinical history and getting vital signs recorded. Adolescents returned after 72 h for the reading of their TST results. Late TST readings were allowed up to a maximum of 7 days. All adolescents were offered HIV counselling and testing during the TST reading day. Parental/ guardian consent and adolescent assent were obtained prior to conducting HIV testing. Adolescents identified with HIV were referred to HIV care and treatment services at the Patient Support Centre at the Siaya District Hospital or to the nearest accessible Patient Support Centre.

The TST cut off we used as evidence for LTBI was ≥10 mm in adolescents that did not have HIV or whose HIV status was unknown and ≥ 5 mm in adolescents with HIV [12].

### Statistical analysis

Statistical analysis was performed using SAS 9.2 (SAS Institute Inc., Cary, NC, US). A three point moving average was used on the TST induration data to look for digit preference by observing differences in peaks between actual and smoothed data. The point prevalence of LTBI and 95% confidence intervals were calculated using survey procedures in SAS and adjusted for clustering. The prevalence of LTBI was estimated using the cut-off method and mirror method [13]. The cut-off method used the study defined cut-off of 10 mm. In the mirror method, the mode of the distribution of those with presumed tuberculous infection is derived from the overall frequency distribution of TST reactions among the adolescents. Then, the total number of adolescents with positive TST reactions was estimated as the sum of the number of adolescents showing reaction sizes equal to the mode and double the number of adolescents with reaction sizes larger than the mode [13]. Bivariate analysis was used to determine significant associations between various risk factors and a positive tuberculin skin test. Factors significant at the *p* < 0.2 level were further explored in a multivariate model using logistic regression. ARTI was calculated with the formula ARTI = 1-(1-prevalence of infection) ^1/mean age^ [14].

## Results

**Fig. 1:**
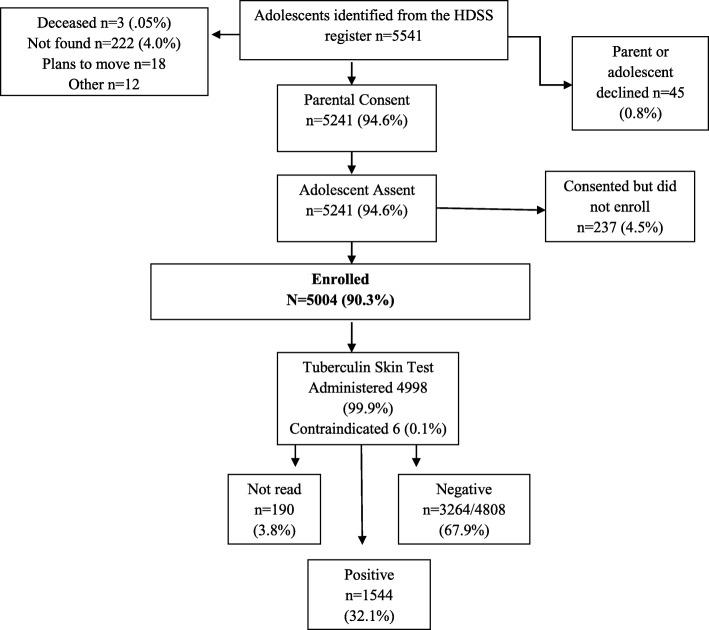
Participant enrolment and tuberculosis skin testing

A total of 5004 adolescents aged 12–18 years were enrolled between August 2008 and August 2009 (Fig. 1). All adolescents enrolled received tuberculin skin tests (TST) and a total of 4808 (96.0%) came for their TST readings and were included in the analysis. There was no difference in socio demographic factors between adolescents dropping after enrolment and before TST readings and those that continued the study. Of the 4808 with TST readings, the mean age was 14.4 (SD 1.9), 2327 (48%) were female, the mean body mass index (BMI) was 17.9 (SD 2.5), 4518 (94%) were currently enrolled in school, 4550 (95%) lived in a rural area, 1289 (26.8%) were orphaned, 861 (18%) had no BCG scar, 23(0.5%) were HIV positive, and 21(0.4%) gave a history of previous tuberculosis (Table 1). There was no significant difference in the baseline characteristics between those who came back for TST readings and those that failed to turn up [data not shown].

**Table 1:** Demographic characteristics of 4808 adolescents undergoing tuberculin skin testing in Kenya, 2008–2009

Characteristic	Category	All (4808)
Tuberculin skin test (TST)	Reactive	1544 (32.1%)
	Non reactive	3264 (67.9%)
Sex	Female	2327 (48.4%)
	Male	2481 (51.6%)
School going	Yes	4518 (94.0%)
	No	290 (6.0%)
HIV status	Positive	23 (0.5%)
	Negative	1985 (41.3%)
	Unknown	2165 (45.0%)
Residence	Rural	4550 (94.6%)
	Urban	258 (5.4%%)
Previous TB	Yes	21 (0.4%)
	No	4787 (99.6%)
History of TB contact	Yes	138 (2.9%)
	No	4670 (97.1%)
Deceased parents	One parent deceased or both alive	3519 (73.2%)
	Both deceased	1289 (26.8%)
Socio economic status	Upper class	1582 (32.9%)
	Middle class	1636 (34.0%)
	Lower class	1590 (33.1%)

### TST indurations

**Fig. 2:**
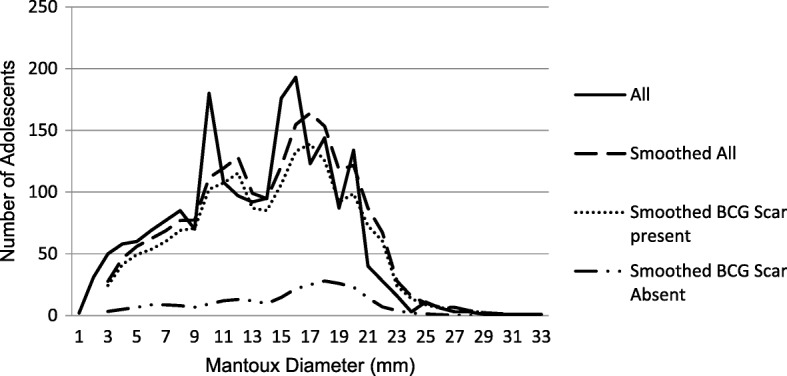
A three point moving average of TST indurations for all adolescents with and without a BCG scar.*BCG scar present 2177/3947(55.2%) had 0 mm induration. *BCG scar absent 585/861 (86%) had 0 mm induration

Of the 4808 adolescents with TST results, 4166 (86.7%) were read within 4 days, 2762 (58%) did not have any induration. Of the 3947 adolescents with a BCG scar, 2177 (55%) had no induration compared to 585/861 (86%) of adolescents without a BCG scar. Among adolescents with TST reactivity, the mean TST induration was 13.2 mm (SD 5.4). There was marked digit preference at 10 mm, 15 and 16 mm and some digit avoidance at 13 mm (Fig. 2). The smoothed data (three-point moving average) of all the adolescents showed a mode of 17 (Fig. 2). Smoothed data for adolescents with and without a BCG scar showed a mode of 17 and 18 respectively (Fig. 2).

### Prevalence of infection and ARTI

Using the cut-off method, including one adolescent with HIV with a TST of 5 mm, the overall prevalence of LTBI was 1544/4808 (32.1, 95% CI 29.2–35.1) with a corresponding ARTI of 2.6% (95% CI 2.2–3.1). Among adolescents with a BCG scar, the prevalence of positive reactions was 33.5% (95% CI 30.3–36.8) with an estimated ARTI of 2.7% (95% CI 2.2–3.2) and among adolescents without a BCG scar, the prevalence of positive reactions was 25.6% (95% CI 22.0–29.2) and the corresponding ARTI 2.1% (95% CI 1.1–3.1) (Table 2). The Mirror method suggested an ARTI of 2.2% (95% CI 1.7–2.7) in those with and 2.1% (95% CI 1.1–3.1) for those without a BCG scar.

**Table 2:** ARTI estimates and prevalence of infection presented for all adolescents, those with and without a BCG scar

	N	Mean age (years)	≥ 10 mm^b^			Mirror method 17 mm^a^		
			*P*	ARTI	CI (95%)	*P*	ARTI^c^	CI (95%)
All	4808	14.4	32.1%	2.6%	(2.2,3.1)	27.6%	2.2%	(1.8,2.6)
BCG scar	3947	14.4	33.5%	2.7%	(2.2,3.2)	27.8%	2.2%	(1.7,2.7)
BCG scar absent	859	14.2	25.6%	2.1%	(1.1,3.1)	27.0%	2.1%	(1.1,3.1)

*CI* 95% Confidence interval

^a^The total number of adolescents with true reactions was calculated by adding the number of adolescents showing reaction sizes equal to the mode to double the number with reaction sizes larger than the mode to determine the numerator

^b^The Cut-off for HIV+ was ≥5 mm

^c^ARTI formula =1-(1-prevalence of infection) ^1/mean age^

### Risk factors for tuberculous infection (LTBI)

Using the cut-off method, risk factors for a positive TST result were being male (OR 1.3, 95% CI 1.2,1.5), history of having a household TB contact (OR 1.5, 95% CI 1.2,1.8), having a BCG scar (OR 1.5,95% CI 1.2,1.7) living in a rural area (OR 1.4, 95% CI 1.1,1.9), and being out of school (OR 1.8,1.4,2.3). The mirror method identified being male (OR 1.4, 95% CI 1.2,1.7) and having a household TB contact (OR 1.6, 95% CI 1.2,2.1) as risk factors for a positive TST (Table 3).

**Table 3:** Risk factors for a positive tuberculin skin test (TST) for the 10 mm cut-off method and the 17 mm mirror method

Risk Factor		Tuberculin skin test > = 10 mm *n* = 4808			Multivariate	Tuberculin skin test > = 17 mm *n* = 4808			Multivariate
		Negative	Positive	OR, 95% CI	OR, 95% CI	Negative	Positive	OR, 95% CI	OR, 95% CI
Sex	Female	1650	677/2327 (29.1%)	1		2076	251/2327 (10.8%)	1	
	Male	1614	867/2481 (34.9%)	1.3 (1.2,1.5)	1.3 (1.2,1.5)	2129	352/2481 (14.2%)	1.4 (1.2,1.6)	1.4 (1.2,1.7)
History of a house hold TB contact	Yes	224	150/374 (40.1%)	1.5 (1.2,1.8)	1.5(1.2,1.8)	119	19/138 (13.8%)	1	
	No	3040	1394/4434 (31.4%)	1		4086	584/4670 (12.5%)	1.6 (1.2,2.1)	1.6(1.2,2.1)
*Previous TB*	Yes	15	6/21 (28.6%)	1		18	3/21 (14.3%)	1	
	No	3232	1528/4787 (32.1%)	0.8 (0.3,2.1)		4163	597/4760 (12.5%)	1.2 (0.4,3.7)	
*BCG Scar*	Yes	2623	1324/3947 (33.5%)	1.3 (1.2,1.7)	1.5 (1.3,1.8)	3450	497/3947 (12.6%)	1.0 (0.8,1.3)	1.0 (0.8,1.3)
	No	641	220/861 (25.6%)	1		753	106/859 (12.3%)	1	
Residence	Urban	189	69/258 (26.7%)	1		231	27/258 (10.5%)	1	
	Rural	3075	1475/4550 (32.4%)	1.2 (1.0,1.5)	1.4 (1.1,1.9)	3974	576/4550 (12.7%)	1.2 (0.8,1.9)	1.2(0.8,1.9)
School going	Yes	3103	1415/4518 (31.3%)	1		3960	558/4518 (12.4%)	1	
	No	161	129/290 (44.5%)	1.8(1.4,2.2)	1.8 (1.4,2.3)	245	45/290 (15.5%)	1.3 (0.9,1.8)	1.3(1.0,1.8)
Orphaned	Yes	1136	585/1721 (34.0%)	1		1474	247/1721 (14.4%)	1	
	No	2128	959/3087 (31.1%)	1.1 (1.0,1.3)	1.1(1.0,1.3)	2731	356/3087 (11.5%)	1.3 (1.1,1.5)	1.3(1.1,1.5)

All risk factors identified during bivariate analysis remained independently associated with a positive TST in multivariate analysis (Table 3).

## Discussion

Our study showed an ARTI of 2.6% (95% CI 2.2–3.1) with the cut-off method and 2.2% (95% CI 1.8–2.6) with the mirror method, both of which are more than double the expected based on serial TST surveys among children aged 10 years in the same area that reported an ARTI of 1.1% [5–7]. This suggests that TB transmission may be more intense among adolescents than among young children. This is consistent with findings by Dodd et al. who suggested estimates of TB infection based on surveys in children may underestimate infection incidence in adults [15]. A higher risk of infection among adolescents than among children of primary school age has also been reported elsewhere [16, 17]. In addition, a golden age has previously been described where the incidence of infection goes down after 5 years of age and begins to rise in adolescence [18].

There are methodological challenges to measuring ARTI in adolescents, including the influence of previous BCG vaccination and exposure to environmental mycobacteria on TST results [19, 20]. Even though TST is a relatively inexpensive test, it has low specificity causing false positives in patients with history of BCG vaccination and environmental mycobacteria exposure [21]. In this study, BCG vaccination status was indeed associated with a positive TST result in the cut-off method, but the mirror method appeared to have eliminated this bias. ARTI was high both in those with and without a BCG scar, using either the cut-off or mirror method. Exposure to environmental mycobacteria appears not to have been a major factor given the low frequency of intermediate reactions (5–9 mm) and the similar prevalence estimate using the cut-off method and mirror method in those without BCG vaccination scar. Even though an interferon gamma release assay (IGRA) was not performed to ascertain TB infection, excellent concordance between tuberculin skin test and IGRA conversion rates has been previously demonstrated [22].

Risk factors for a positive TST were similar to those reported in studies elsewhere, including male sex [9] and having a BCG scar [23]. The higher risk of TB infection in males might be related to older boys having more contact with adult men among whom TB prevalence is higher, as suggested by Dodd [15]. Although only a small proportion (6%) of adolescents in this study were not currently enrolled in school, they were significantly more likely to have a positive TST compared to in school youth 45 and 31% respectively (OR 1.8, 95% CI 1.1, 2.9). However, this subgroup may be a good target population for intensified case finding and prevention. The majority of adolescents enrolled were of rural residence (94.6%) and this was associated with a positive TST also in a multivariate model. However, the difference was not large (32% versus 27%). As expected history of a household contact was strongly predictive of a positive TST 40.1% versus 31.4%). Previous TB surprisingly was not associated with a positive TST, but numbers were small. Having both parents deceased was associated with a positive TST (34% versus 31%)). The study area has a high HIV prevalence of 15.1% [2] and many deaths among parents are likely to have been due to HIV/AIDS, which in this area is strongly associated with tuberculosis indicating a higher risk of LTBI in orphaned children before their parents died.

The ARTI estimates are derived from a point prevalence estimate and repeated TST surveys would need to be conducted to see how this correlates with the incidence of infection and age associated risks of infection [16, 17]. While BCG had some influence on TST reading results using the cut-off method, this was no longer the case using the mirror method. Digit preference was corrected by smoothing, though might be further reduced in the future by further strengthening training and supervision.

Preventing TB disease instead of infection has been the key goal of vaccine development [24]. Epidemiological and mathematical modelling studies have shown a pre-infection vaccine would have a high level impact on TB disease control [24]. A pre-infection vaccine will require choice of an age range where the risk of infection rises but before majority of the age group becomes infected. Adolescents are an important group for vaccination against TB because while TB incidence is relatively low in the age group 5–14 years, it rises rapidly in adolescence [25]. In our study majority of adolescents (67.9%) were TST negative indicating this would be a good age group to target for prevention of infection vaccine studies. We have reported on the incidence of tuberculosis in adolescents elsewhere [26]. The incidence of TB addresses the sample sizes needed for TB vaccine efficacy trials preventing disease; whether primary disease or reactivation from previous TB infection. Because of difficulties in having a human challenge model with tuberculosis, a pathway to understanding the immune mechanisms of protection against tuberculosis with new vaccines would be to conduct prevention of infection with *M.tuberculosis* studies as a marker of biologic impact [24]. The studies will need to take into account the huge burden of non tuberculous mycobacteria (NTM) infection in this population including using interferon gamma release assays to disciminate M.TB infection from NTM infection [11]. Since infection with *M.tuberculosis* happens much more often than TB disease, the trials will be much smaller and trial results would be obtained much sooner allowing a read-out on a TB vaccine candidate’s likely efficacy before doing larger scale efficacy studies.

## Conclusion

We conclude that the high TB transmission rates we found in this study, suggest that adolescents in schools in this region may be an appropriate target group for TB vaccine trials including TB vaccine trials aiming to prevent infection. Out of school adolescents might be harder to enroll and follow up for TB incidence.

## Additional file

Additional file 1: Annual risk of TB infection (ARTI). The data set provides all the variables that were used for analysis of the results in this manuscript. (CSV 1400 kb)

## Data Availability

The data supporting the findings of this study have been included as an additional supporting file in the submission (Additional file 1).

## Abbreviations

ARTI: Annual Risk of tuberculous infection
BCG: Bacille Calmette-Guerin
HDSS: Health and demographic surveillance system
HIV: Human immunodeficiency virus
LTBI: Latent tuberculosis infection
MTB: *Mycobacterium tuberculosis*
POI: Prevention of infection
PPD: Purified protein derivative
TB: Tuberculosis
TST: Tuberculin skin test
TU: Tuberculin units
